# A new family of siphonostomatoid Copepoda (Pupiformidae fam. nov.) from the Korea Strait

**DOI:** 10.3897/zookeys.1284.194182

**Published:** 2026-07-08

**Authors:** Jimin Lee, Il-Hoi Kim

**Affiliations:** 1 Ocean Climate Response & Ecosystem Research Department, Korea Institute of Ocean Science & Technology, Busan 49111, Republic of Korea Ocean Climate Response & Ecosystem Research Department, Korea Institute of Ocean Science & Technology Busan Republic of Korea; 2 Korea Institute of Coastal Ecology, Bucheon 14449, Republic of Korea Korea Institute of Coastal Ecology Bucheon Republic of Korea

**Keywords:** Associated copepods, new species, *Pupiformus* gen. nov., taxonomy

## Abstract

Two new species of *Pupiformus***gen. nov**., *P. apodus***sp. nov**. and *P. rugosus***sp. nov**., belonging to the copepod order Siphonostomatoida, are described based on specimens collected from washings of benthic animals (invertebrates and fish) dredged from the Korea Strait at a depth of 116–123 m. *Pupiformus***gen. nov**. is characterized by having a 6-segmented, pupiform body, an unilobed maxillule, and a cephalothoracic shield divided into a dorsal tergite and two lateral pleurites, as well as by lacking legs, maxillae, and a mandibular stylet. *Pupiformus rugosus***sp. nov**. differs from *P. apodus***sp. nov**. in having a smaller body (about 500 μm long, compared to about 900 μm in *P. apodus***sp. nov**.), rugose dorsal tubercles on the cephalothorax and metasomites (vs smooth tubercles in *P. apodus***sp. nov**.), a 5-segmented antennule (vs 4-segmented in *P. apodus***sp. nov**.), two long apical setae on the mandibular palp (vs three setae in *P. apodus***sp. nov**.), a 4-segmented female maxilliped (vs 3-segmented in *P. apodus***sp. nov**.), and by lacking caudal rami (present in *P. apodus***sp. nov**.). Since the new genus cannot be assigned to any existing family of the Copepoda, a new family-level taxon, Pupiformidae**fam. nov**., is established to accommodate it.

## Introduction

Copepoda is the largest and most diverse group of crustaceans (Vakati et al. 2023), comprising 14,485 species that exhibit a wide range of morphological and lifestyle variations (Bernot et al. 2021). The copepod order Siphonostomatoida Burmeister, 1835 includes 2,262 known species (Bernot et al. 2021) and contains parasitic families like the Caligidae Burmeister, 1835, Lernaeopodidae Milne Edwards, 1840, and Pennellidae Burmeister, 1835, which are often found on fish. The siphonostomatoid families, Asterocheridae Giesbrecht, 1899, Cancerillidae Giesbrecht, 1897, and Nicothoidae Dana, 1852–1853, are known to be symbionts of marine invertebrates such as sponges, echinoderms, and corals.

The Korea Strait is a sea passage between the Korean Peninsula and Japan in East Asia. It has an average depth of 90–100 m and a maximum depth of about 230 m (Park et al. 2000). The climate of this region is strongly influenced by the warm Kuroshio Current, which carries heat, marine larvae, and nutrients into the Korean Strait and thereby supports a biologically rich subtropical or warm-temperate ecosystem that sustains a highly diverse marine life (Jang et al. 2012).

During a recent exploration of Korea strait, the Korea Institute of Ocean Science and Technology dredged benthic invertebrates and fish. An examination of washings from this material revealed several new species of symbiotic copepods, some of which do not belong to any existing families. This paper describes two new species of siphonostomatoid copepods and establishes a new family to accommodate them.

## Materials and methods

Copepod samples examined in this study were collected from washings of benthic animals, including invertebrates and fish, dredged at a depth of 116–123 m in the Korea Strait, east of Jeju Island (33°30'N, 128°46'E). The collected copepods were fixed with formalin for several hours and then preserved in 80% ethanol. Before microscopic observation, selected copepod specimens were immersed in lactic acid for at least 10 min. Dissection was performed using the reversed slide method of Humes and Gooding (1964). Drawings were made with a drawing apparatus attached to a microscope. The lengths of appendage segments are calculated using the averages of the longest and shortest margins. For observations using scanning electron microscopy, samples were dehydrated through a graded ethanol series from 50% to 100% in 10% steps, three times for 10 min at each step, then transferred to hexamethyldisilazane and air-dried. After carbon coating (MC1000; Hitachi, Tokyo, Japan), the samples were examined using a scanning electron microscope (S-4300; Hitachi, Tokyo, Japan) at 10 kV. Type specimens have been deposited at the Marine Biodiversity Institute of Korea (**MABIK**), Seocheon, Korea.

## Systematic accounts

**Class Copepoda Milne Edwards, 1840**

**Order Siphonostomatoida Burmeister, 1835**

### Pupiformidae fam. nov.

Taxon classificationAnimaliaCopepodaSiphonostomatoida

C6209459-6522-568D-9C1F-4BB86E22A615

https://zoobank.org/E867833-2515-41F9-A7D4-6A27F067C79C

#### Diagnosis.

As for the type genus.

#### Type genus.

*Pupiformus* gen. nov.

### Pupiformus gen. nov.

Taxon classificationAnimaliaSiphonostomatoidaPupiformidae

6BC5E53C-6B1E-55B0-B0C6-98B7D7A9274A

https://zoobank.org/E231E2E0-E64F-4E2E-BCB9-B30B7BBFE592

#### Diagnosis (female).

Body pupiform, cylindrical, 6-segmented, consisting of cephalothorax, 4-segmented metasome, and genitoabdomen. Cephalothoracic shield clearly divided into dorsal tergite and 2 lateral pleurites. Metasome comprising second to fifth pedigerous somites. Genitoabdomen lacking any trace of segmentation; genital apertures positioned ventrally. Caudal rami absent or rudimentary. Rostrum developed. Antennule short, 4- or 5-segmented; terminal segment bearing 1 aesthetasc on proximal anterior margin. Antenna 4-segmented, consisting of coxa, basis, and 2-segmented endopod; exopod absent; second endopodal segment bearing small claw distally. Oral cone short, but distinct, comprising anterior and posterior lips (labrum and labium). Mandible represented by 1-segmented palp; stylet absent. Maxillule 2-segmented; proximal segment (precoxa or pedetal) unarmed; distal segment (palp or fusion of inner and outer lobes) with 3 setae (2 apical and 1 proximal). Maxilla absent. Maxilliped 3- or 4-segmented, consisting of coxa, basis, and 2-segmented endopod, or coxobasis and 2-segmented endopod; second endopodal segment bearing claw at tip. Legs 1–4 vestigial, represented by large sclerotized pads. Legs 5 and 6 absent.

#### Etymology.

The generic name refers to the pupiform body shape of the species within the genus. Gender masculine.

#### Type species.

*Pupiformus apodus* sp. nov. (original designation).

#### Other included species.

*Pupiformus rugosus* sp. nov.

### Pupiformus apodus sp. nov.

Taxon classificationAnimaliaSiphonostomatoidaPupiformidae

81DBED1A-73E1-529A-8896-8F9CEA372381

https://zoobank.org/47D565CE-B3A5-4F77-AAA7-FE7E8CD1DEF5

Figs 1, 2

#### Type locality.

Korea Strait, east of Jeju Island (33°30'55"N, 127°46'49"E), depth 116–123 m.

#### Type material.

Holotype (intact ♀, MABIK CR00261359) and paratypes (6 intact ♀♀, MABIK CR00261360; 2 dissected ♀♀) from washings of benthic animals dredged from the Korea Strait, collected by J. Lee, 20 August 2025. Intact type specimens have been deposited in the Marine Biodiversity Institute of Korea (MABIK), Seocheon, Korea. Dissected paratypes are kept in the collection of I.-H. Kim.

#### Etymology.

The name is derived from the Greek words *a* (= without) and *pod* (= a foot), referring to the absence of legs in the new species.

#### Description.

**Figure 1.**
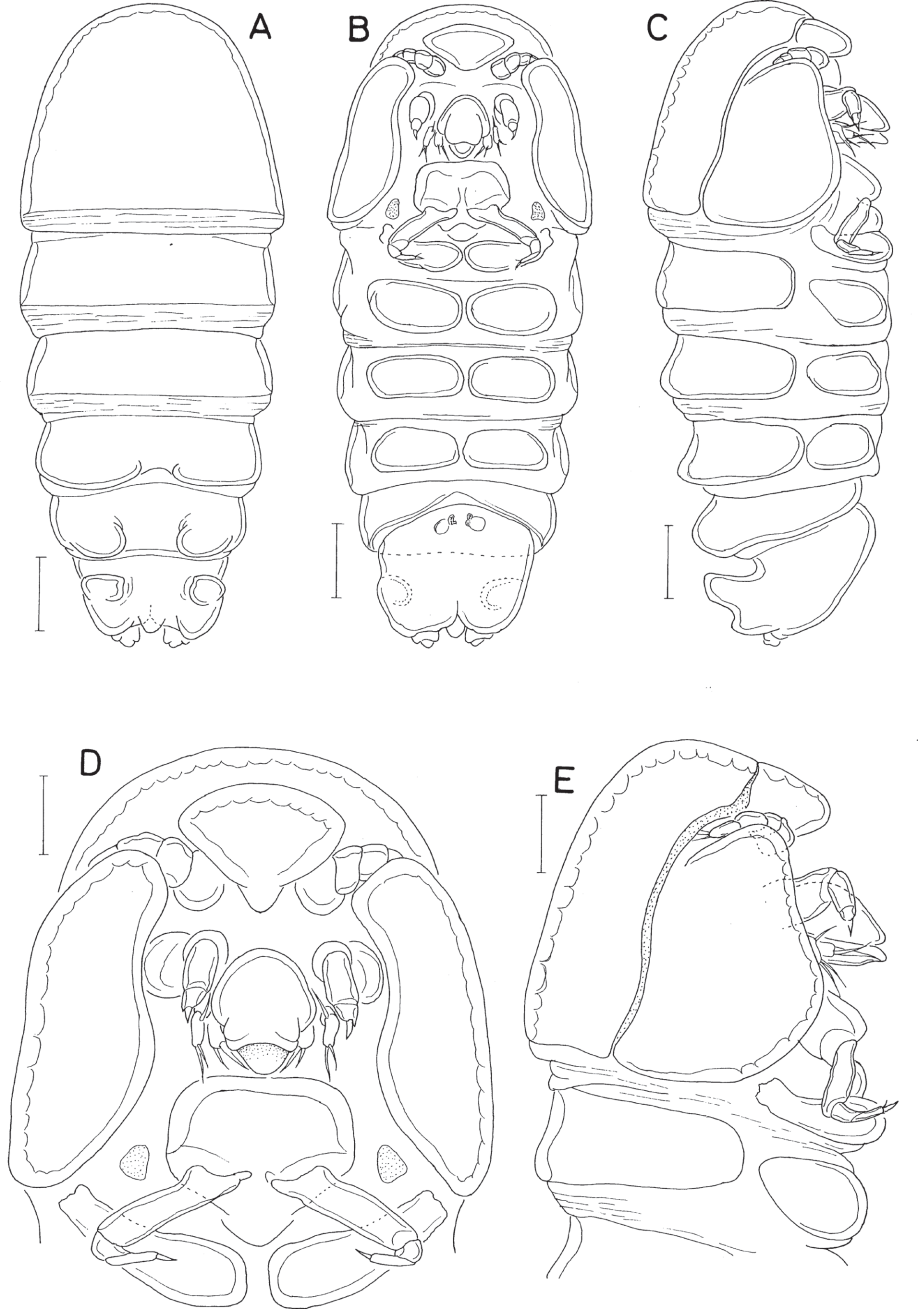
*Pupiformus apodus* gen. et sp. nov., female. **A**. Habitus, dorsal; **B**. Habitus ventral; **C**. Habitus, right; **D**. Cephalothorax, ventral; **E**. Cephalothorax and first pedigerous somite, right. Scale bars: 0.1 mm (**A–C**); 0.05 mm (**D**); 0.07 mm (**E**).

**Figure 2.**
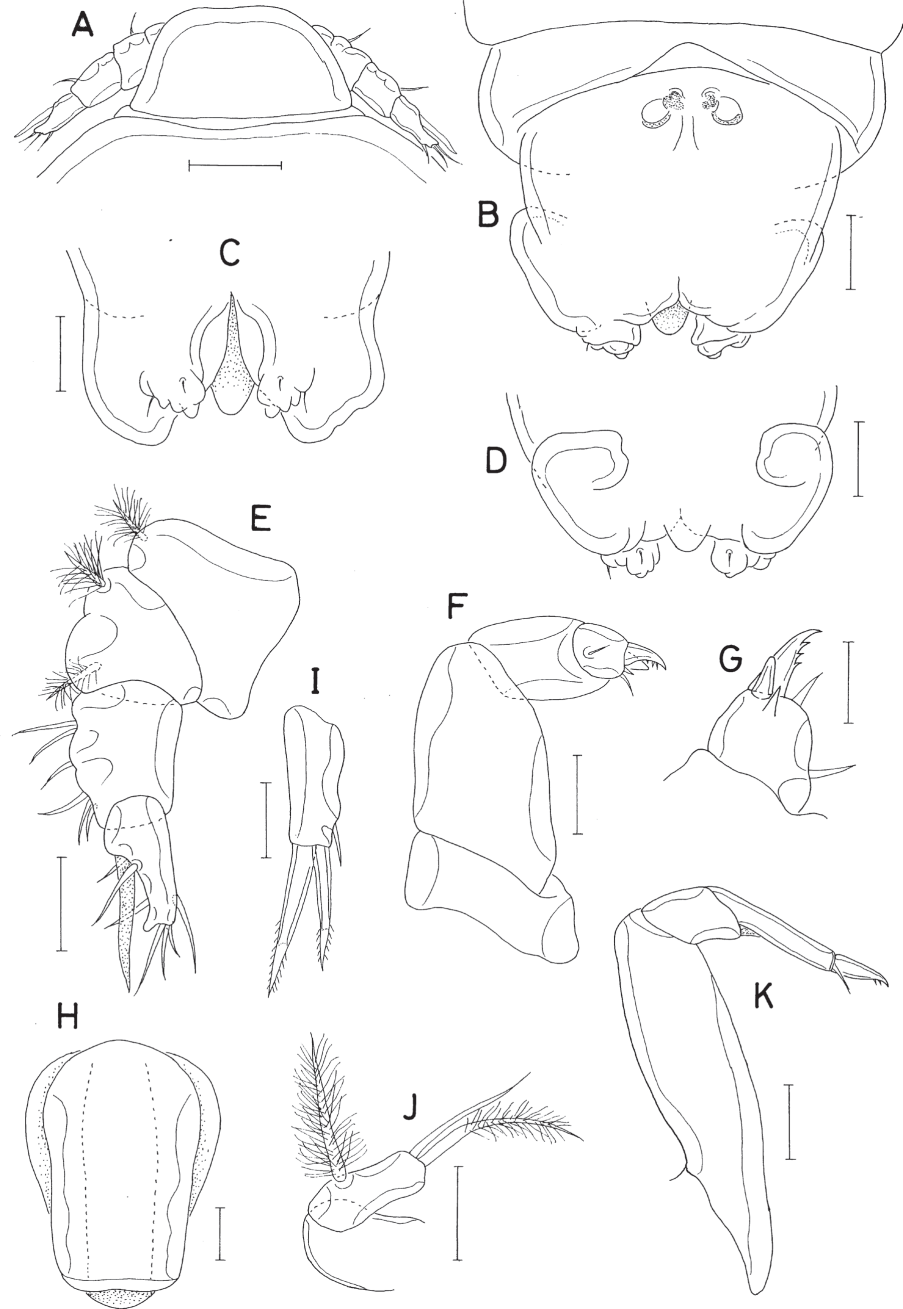
*Pupiformus apodus* gen. et sp. nov., female. **A**. Rostral area, anterior; **B**. Posterior part of body, ventral; **C**. Posterior part of genitoabdomen, ventrodistal; **D**. Posterior part of genitoabdomen, dorsal; **E**. Antennule; **F**. Antenna; **G**. Distal part of antenna; **H**. Oral cone, anterior; **I**. Mandible; **J**. Maxillule; **K**. Maxilliped. Scale bars: 0.05 mm (**A–D**); 0.02 mm (**E, F, H–K**); 0.01 mm (**G**).

**Female**. Body (Fig. 1A–C) pupiform, cylindrical, appearing immovable, 6-segmented, consisting of cephalothorax, metasome, and genitoabdomen, with thick, hard integument. Body length 850–970 μm, based on 7 specimens. Body length of figured and described specimen 895 μm. Maximum width 367 μm across cephalothorax. Cephalothorax 324 μm long, wider than long. Cephalothoracic shield clearly divided into 3 parts (dorsal tergite and 2 lateral pleurites) by 2 longitudinal, unsclerotized grooves (Fig. 1C, E). Metasome 4-segmented, comprising second to fifth pedigerous somites; sizes of these somites 145 × 356, 116 × 360, 102 × 313, and 90 × 273 μm, respectively, from anterior to posterior. Fourth metasomite with tubercle on each dorsolateral side. Genitoabdomen (Fig. 2B) with tubercle on each dorsolateral side (Fig. 2D), distinct anal operculum (Fig. 2B–D); genital apertures (Fig. 2B) positioned at proximal region of ventral surface. Caudal rami (Fig. 2C) as small rugose distal process on genitoabdomen; each caudal ramus with 3 indistinct, minute setae.

Rostrum (Figs 1D, 1E, 2A) well developed, with bluntly tapering distal apex. Antennule (Fig. 2E) short, 53 μm long, 4-segmented; armature formula 1, 2, 6, and 7+aesthetasc; three setae on first and second segments plumose, other setae naked; terminal segment consisting of broader proximal third and narrowed distal two-thirds, its aesthetasc pointed distally, as long as segment. Antenna (Fig. 2F) stout, 4-segmented, consisting of coxa, basis, and 2-segmented endopod; first to third segments unarmed; last segment (second endopodal segment; Fig. 2G) short, wider than long, with terminal claw bearing 3 teeth on concave margin, 1 blunt distal spine, and 3 setae (2 distal and 1 proximal).

Oral cone (Figs 1D, 1E, 2H) short, but distinct, 102 × 75 μm in anterior view. Mandible (Fig. 2I) consisting of 1-segmented palp, 38 × 16 μm, bearing 2 thick, distal setae of 41 and 34 μm long, respectively, and 1 small subdistal, naked seta of 11 μm long; mandibular stylet absent. Maxillule (Fig. 2J) 2-segmented; proximal segment unarmed, not articulated from ventral surface of cephalothorax; distal segment 24 × 12 μm, bearing 3 setae (1 plumose proximal seta, and 1 plumose and 1 naked distal setae). Maxilla absent, but its vestige presents as sclerotized spot positioned lateral to base of maxilliped (Fig. 1D). Maxilliped (Fig. 2K) 3-segmented, consisting of coxobasis and 2-segmented endopod; second endopodal segment slender, 30 μm long, twice longer than first endopodal segment, distally bearing 1 small seta and 1 claw; latter claw 15 μm long, bearing 2 denticles subdistally.

Leg 1–4 vestigial, represented by large, sclerotized pads (Fig. 1B, C). Legs 5 and 6 absent.

**Male**. Unknown.

#### Remarks.

The division of the cephalothoracic shield of *P. apodus* sp. nov. into the dorsal tergite and two lateral pleurites is clearly defined and consistent in all examined specimens. This characteristic also appears in *P. rugosus* sp. nov. described below.

### Pupiformus rugosus sp. nov.

Taxon classificationAnimaliaSiphonostomatoidaPupiformidae

8EB438B2-C52D-5EBF-8B02-3E5B7F3835A1

https://zoobank.org/7754AD10-83E1-4559-BAC3-E633679AD193

Figs 3, 4, 5

#### Type locality.

Korea Strait, east of Jeju Island (33°30'55"N, 127°46'49"E), depth 116–123 m.

#### Type material.

Holotype (intact ♀, MABIK CR00261361) and paratypes (11 intact ♀♀, MABIK CR00261362; 2 dissected ♀♀) collected with *Pupiformus apodus* sp. nov. from washings of benthic animals dredged from the same locality as the former species. Intact type specimens have been deposited in the Marine Biodiversity Institute of Korea (MABIK), Seocheon, Korea. Dissected paratypes are kept in the collection of I.-H. Kim.

#### Etymology.

The name is an adjective (Latin: *rugosus*) and refers to the rugose dorsal tubercles of the new species.

#### Description.

**Figure 3.**
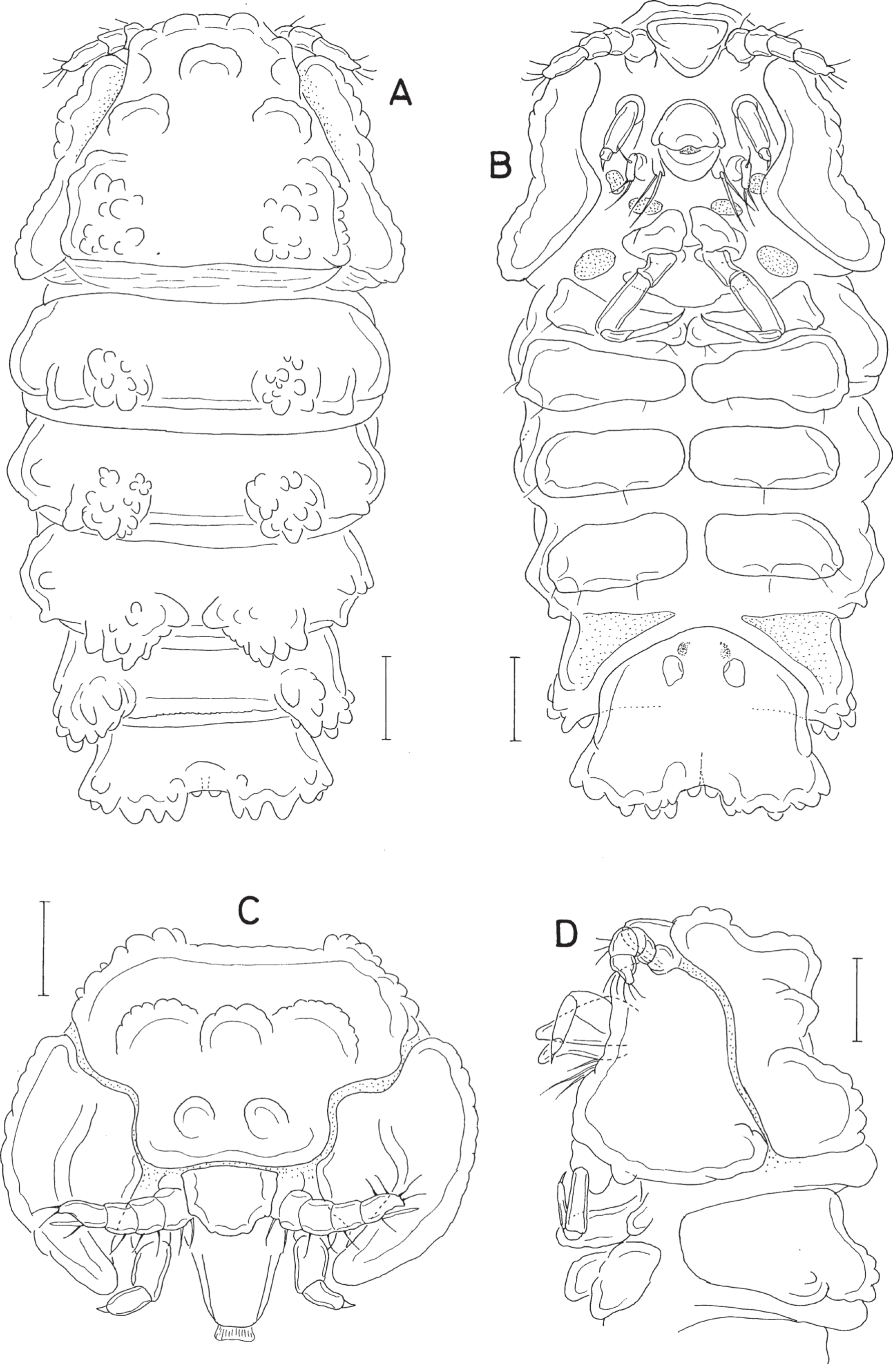
*Pupiformus rugosus* gen. et sp. nov., female. **A**. Habitus, dorsal; **B**. Habitus, ventral; **C**. Cephalothorax, anterior; **D**. Cephalothorax and first pedigerous somite, left. Scale bars: 0.05 mm.

**Female**. Body (Figs 3A, 3B, 5) shaped and segmented as in *P. apodus* gen. et sp. nov. Body length of figured and described specimen 501 μm. Mean body length 502 μm, based on 7 specimens. Maximum width 242 μm. Cephalothorax 180 × 242 μm, gradually broadened posteriorly, consisting of clearly divided dorsal tergite and lateral pleurites (Fig. 3C, D); tergite bearing 5 tubercles (arranged as 2, 1, and 2) on anterior half and large posterolateral tubercles each bearing about 10 irregularly arranged, wart-like processes (Fig. 3A, C). Ventral surface of cephalothorax bearing 3 pairs of sclerotized areas: one on lateral side of maxillule, another between oral cone and maxilliped, and the other lateral to maxilliped. Pleurites with uneven lateral and ventral margins (Fig. 3A, C). Metasomites each with pair of tubercles at dorsal or dorsolateral tubercles bearing about 10 or more globular processes. Fourth metasomite with sclerotized region on both sides of ventral surface (Fig. 3B). Genitoabdomen 105 × 160 μm in ventral view, with several processes on each side of posterior margin, small, excavated anal region bearing pair of small lobes (Fig. 3A, B); genital apertures positioned proximal region of ventral surface; anal operculum absent. Caudal rami absent.

**Figure 4.**
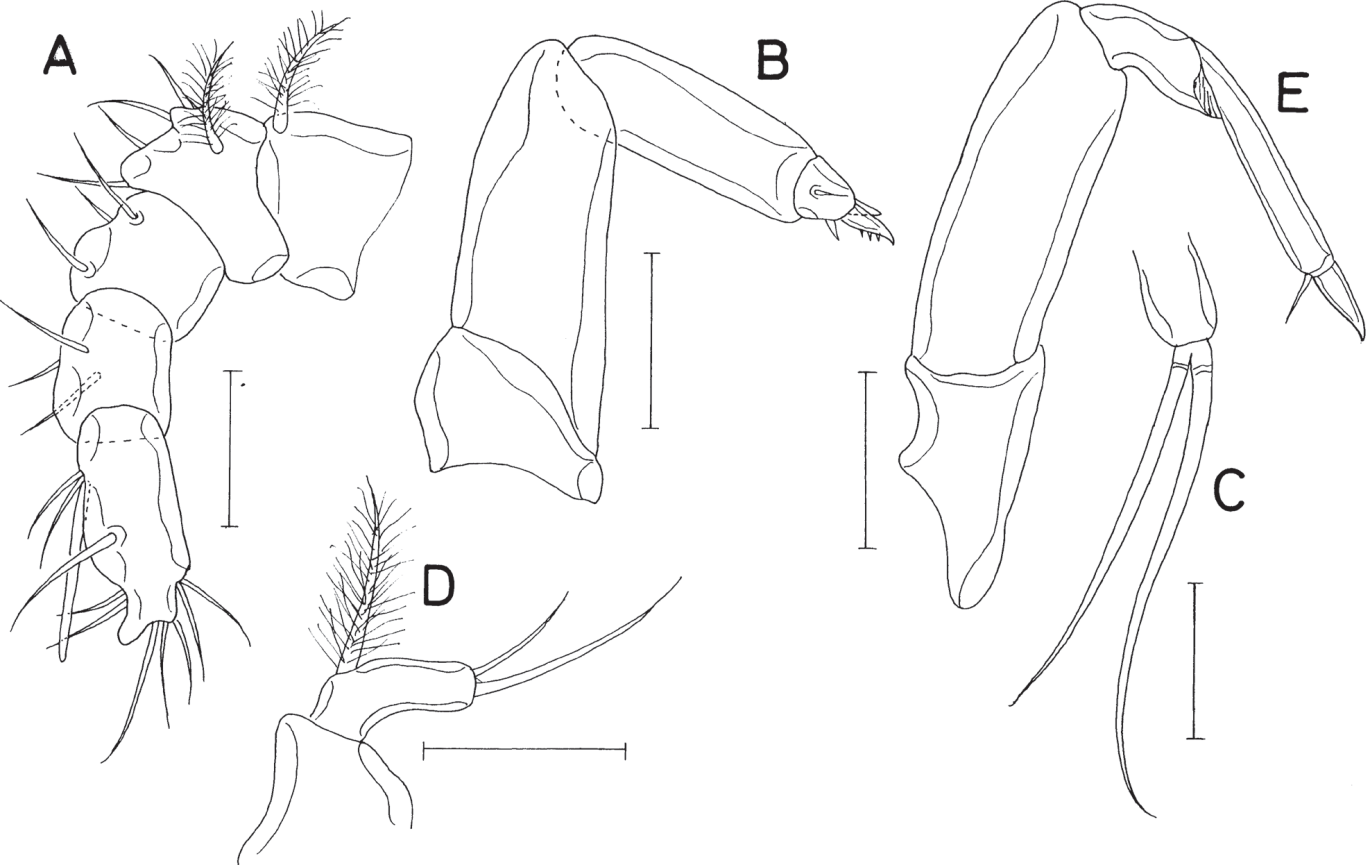
*Pupiformus rugosus* gen. et sp. nov., female. **A**. Antennule; **B**. Antenna; **C**. Mandible; **D**. Maxillule; **E**. Maxilliped. Scale bars: 0.02 mm.

Rostrum (Fig. 3B) roughly trapezoid in anterior view (Fig. 3C), well separated from tergite. Antennule (Fig. 4A) small, 42 μm long, 5-segmented; armature formula 1, 5, 3, 3, and 10+aesthetasc; seta of first segment and first seta of second segment plumose, other setae naked; aesthetasc on last segment extending slightly over distal end of terminal segment; terminal segment bearing produced, nipple-like anterodistal corner. Antenna (Fig. 4B) 4-segmented, consisting of coxa, basis, and 2-segmented endopod; lengths of segments 11, 33, 30, and 7 μm, respectively, from proximal to distal; terminal segment (second endopodal segment) short, bearing 1 claw bearing 3 denticles, 1 small spine, and 2 small setae.

Oral cone (Fig. 3C) distinct, bearing membranous apex. Mandible (Fig. 4C) represented by 1-segmented palp bearing 2 naked distal setae (58 and 48 μm long, respectively). Maxillule (Fig. 4D) 2-segmented; proximal segment unarmed; distal segment with 3 setae (plumose proximal seta and 2 naked, unequal distal setae). Maxilla (Fig. 3B) absent. Maxilliped (Fig. 4E) 4-segmented; proximal 3 segments (syncoxa, basis, and first endopodal segments) unarmed; basis and 2 endopodal segments 42, 12, and 26 μm long, respectively; slender second endopodal segment distally bearing small claw (10 μm long) and 1 small seta.

**Figure 5.**
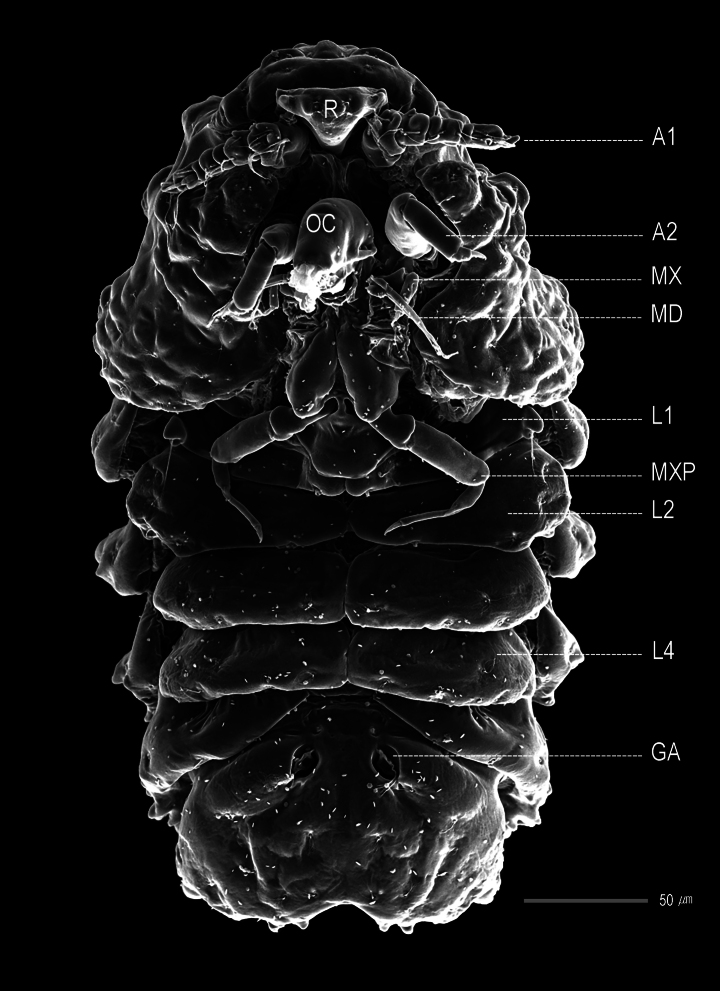
SEM micrograph of *Pupiformus rugosus* gen. et sp. nov., female, ventral. (A1, Antennule; A2, Antenna; GA, Genitoabdomen; L1, Leg 1; L2, Leg 2; L4, Leg 4; MD, Mandible; MX, Maxillule; MXP, Maxilliped; OC, Oral cone; R, Rostrum)

Legs 1–4 vestigial, represented by large, sclerotized pads (Fig. 3B); each pad bearing 1–3 hair-like setules on its lateral and posterior margins. Legs 5 and 6 absent.

**Male**. Unknown.

#### Remarks.

*Pupiformus rugosus* sp. nov. and *P. apodus* sp. nov. are very similar, sharing key diagnostic features of the genus. However, *P. rugosus* sp. nov. differs significantly from *P. apodus* sp. nov. in several female morphological details: (1) the body is smaller, about 500 μm long, compared to roughly 900 μm in *P. apodus* sp. nov.; (2) the cephalothorax and metasomites have rugose dorsal tubercles, whereas in *P. apodus* sp. nov., these structures are either absent or smooth; (3) caudal rami are absent in *P. rugosus* sp. nov., but are present as rudimentary processes in *P. apodus* sp. nov.; (4) the antennule has five segments, vs four in *P. apodus* sp. nov.; (5) the mandibular palp has two long, naked setae at its tip, whereas *P. apodus* sp. nov. has two stout, spiniform distal setae and one small, naked, subdistal seta; and (6) the maxilliped is 4-segmented, vs three segments in *P. apodus* sp. nov.

## Discussion

With a prominent oral cone formed by the anterior labrum and posterior labium, *Pupiformus* gen. nov. clearly belongs to the Siphonostomatoida, a copepod order that currently comprises 40 families (Walter and Boxshall 2026). *Pupiformus* gen. nov. exhibits several unusual morphological features that prevent its assignment to any existing family within the Siphonostomatoida (Table 1). The most striking feature of the new genus is the loss of legs, represented only by pad-like vestiges of legs 1–4. Parasitic siphonostomatoid copepods either lack legs entirely or possess reduced or modified legs. The pad-like leg vestiges observed in *Pupiformus* gen. nov. are extremely rare among the Siphonostomatoida, shared perhaps only with two genera: *Peniculus* von Nordmann, 1832, the most primitive genus within the family Pennellidae (Kabata 1979), and the closely related genus *Peniculisa* C.B. Wilson, 1917. Examples of vestigial pad-like legs were well illustrated by Boxshall (1986) and Venmathi Maran et al. (2012) for *Peniculus* species, and by Uyeno and Nagasawa (2010) for *Peniculisa* species. Unlike *Pupiformus* gen. nov., these fish-parasitic pennellid genera possess a maxilla and a subchelate antenna, and lack a maxilliped, thus diverging from the new genus.

**Table 1.** Distribution of five selected characters in siphonostomatoid families (symbols: +, present; ×, absent; ?, unknown).

	**Characters**				
	**Maxilla**	**Swimming legs**	**Mandibular stylet**	**Mandibular palp**	**Lobes of Maxillule**
Archidactylinidae	+	+	+	×	2
Artotrogidae	+	+	+	×	2
Asterocheridae	+	+	+	+	2
Brychiopontiidae	+	+	+	×	2
Caligidae	+	+	+	×	1
Calverocheridae	+	+	×	×	1
Cancerillidae	+	+	+	×	1, 2
Codobidae	+	+	+	×	2
Coralliomyzontidae	+	+	+	+	2
Dichelesthiidae	+	+	+	×	2
Dichelinidae	+	+	×	+	1
Dinopontiidae	+	+	+	×	2
Dirivultidae	+	+	+	×	2
Dissonidae	+	+	+	×	2
Ecbathyriontidae	+	+	+	×	2
Entomolepididae	+	+	+	+	2
Eudactylinidae	+	+	+	×	2
Hatschekiidae	+	+	+	×	2
Hyponeoidae	+	+	+	×	2
Kroyeriidae	+	+	+	×	2
Lernaeopodidae	+	×	+	×	2
Lernanthropidae	+	+	+	×	2
Megapontiidae	+	+	+	×	2
Micropontiidae	+	+	+	+	2
Nanaspididae	+	+	+	?	2
Nicothoidae	+, ×	+, ×	+	+, ×	2
Pandaridae	+	+	+	×	2
Pennellidae	+	+, ×	+	×	1
Pontoeciellidae	+	+	+	+	1
Pseudocycnidae	+	+	+	×	1
Pseudohatschekiidae	+	+	+	×	1
Rataniidae	+	+	+	+	2
Samarusidae	+	+	+	×	2
Scottomyzontidae	+	+	+	+	2
Sphyriidae	+	×	+	×	2
Sponginticolidae	×	×	×	×	×
Spongiocnizontidae	+	×	+	×	2
Stellicomitidae	+	+	+	+	2
Tanypleuridae	+	×	+	×	1
Trebiidae	+	+	+	×	2
Pupiformidae fam. nov.	×	×	×	+	1

Almost all known siphonostomatoid copepods possess a maxilla, so its absence is another notable feature of *Pupiformus* gen. nov. The maxilla is also missing in members of genus *Fissuricola* Humes, 1987 of the family Dirivultidae Humes & Dojiri, 1981 (Humes 1987), the genus *Sponginticola* Topsent, 1928 of the monotypic family Sponginticolidae Topsent, 1928, and some genera of the family Nicothoidae, such like *Arhizorhina* Bamber & Boxshall, 2006, and *Rhizorhina* Hansen, 1892 (Bamber and Boxshall 2006; Kakui 2016). However, we infer that *Pupiformus* gen. nov. is not related to the Dirivultidae, Sponginticolidae, or Nicothoidae, as copepods of these families exhibit very different morphological features. For example, species of the Dirivultidae possess well-developed legs, while those of the Sponginticolidae and Nicothoidae have highly transformed, unsegmented bodies.

The ancestral form of the siphonostomatoid mandible is observed in the genus *Asterocheres* Boeck, 1859, of the family Asterocheridae (Huys and Boxshall 1990), in which the mandible comprises a stylet-like coxal gnathobase and a palp. The palp is absent in all fish-parasitic siphonostomatoid families (Huys and Boxshall 1990) and in several invertebrate-associated families, including the Artotrogidae Brady, 1880, Brychiopontiidae Humes, 1974, Calverocheridae Stock, 1968, Cancerillidae Giesbrecht, 1897, and Dinopontiidae Murnane, 1967. Conversely, the mandibular stylet is present in nearly all siphonostomatoid families, except for the Calverocheridae, Dichelinidae Boxshall & Ohtsuka, 2001, and Sponginticolidae. Thus, the lack of a mandibular stylet represents a key morphological characteristic of *Pupiformus* gen. nov.

The basic structure of the siphonostomatoid maxillule is bilobed, consisting of an inner lobe representing the precoxal gnathobase and an outer lobe representing the palp (Huys and Boxshall 1990). Both lobes of the maxillule are armed with setae at the apex, similar to those found in the genus *Asterocheres*. Among the Siphonostomatoida, a unilobed maxillule is observed in copepods of eight families: Calverocheridae, Cancerillidae, Dichelinidae, Pennellidae, Pontoeciellidae Giesbrecht, 1895, Pseudocycnidae C.B. Wilson, 1922, Pseudohatschekiidae Tang, Izawa, Uyeno & Nagasawa, 2010, and Tanypleuridae Kabata, 1969 (Boxshall and Ohtsuka 2001; Boxshall and Halsey 2004; Tang et al. 2010). The maxillule of *Pupiformus* gen. nov. appears to be 2-segmented, with an unarmed proximal segment and a distal segment bearing two distal setae and one proximal seta. Among other siphonostomatoid copepods, a similar maxillule form was well illustrated by Venmathi Maran et al. (2012) in their redescription of *Peniculus truncatus* Shiino, 1956. In the redescription of *Pseudohatschekia branchiostegi* Yamaguti, 1939 of the Pseudohatschekiidae, Tang et al. (2010) interpreted the unilobed maxillule bearing three setae as the outer proximal seta representing the palp, and the distal part bearing two setae as the precoxal endite. Therefore, in the maxillules of *Pupiformus* gen. nov. and *Peniculus truncatus*, the proximal segment is actually a pedestal. The distal segment bearing three setae have resulted from the fusion of the inner and outer lobes, and the proximal seta is likely a vestige of the outer lobe. Unlike *Pupiformus* gen. nov., copepods of all of the families mentioned above typically possess a maxilla, legs (except the Tanypleuridae and most species of the Pennellidae), a mandibular stylet (except the Calverocheridae and the Dichelinidae), and lack a mandibular palp (except the Dichelinidae and the Pontoeciellidae). Consequently, *Pupiformus* gen. nov. cannot be assigned to any of these families.

In conclusion, the absence of legs and maxillae, along with the presence of a mandible represented only by a palp and a unilobed maxillule, are key morphological features of *Pupiformus* gen. nov. that prevent its assignment to any known family within the order Siphonostomatoida (Table 1). Therefore, creating a new family, Pupiformidae fam. nov., is necessary to accommodate the new genus. The division of the cephalothoracic shield into a dorsal tergite and two lateral pleurites is consistent in all examined specimens of the two new species of *Pupiformus* gen. nov. Because this feature has not been observed in other siphonostomatoid copepods, it likely provides an additional diagnostic characteristic defining the new family.

Unfortunately, the host of *Pupiformus* gen. nov. and its basic biological traits remain unknown. The loss of the mandibular stylet in Siphonostomatoida is limited to copepods associated with invertebrates; therefore, the new genus, which lacks the mandibular stylet, is likely associated with an invertebrate host. The absence of functional legs and the presence of weak antennules in *Pupiformus* gen. nov. suggest that copepods of this new genus are incapable of free swimming. The absence of the maxilla and underdevelopment of other potential attachment organs (i.e. the antenna and maxilliped) indicate that copepods of this genus are unable to attach firmly to the host. These observations suggest that species of *Pupiformus* gen. nov. inhabit some cavity on the host, such as a gall. Examples of gall-inhabiting siphonostomatoid copepods include *Calverocheres* C.B. Wilson, 1932 species of the Calverocheridae, which have been recorded from galls formed on the spines of echinoids (Stock 1968), as well as the genera *Hammatimyzon*, *Cecidomyzon*, *Oedomyzon*, and *Cystomyzon* of the family Asterocheridae, recorded by Stock (1981) from galls of stylasterine corals.

## Supplementary Material

XML Treatment for Pupiformidae

XML Treatment for Pupiformus

XML Treatment for Pupiformus apodus

XML Treatment for Pupiformus rugosus

## Additional information

### Artificial Intelligence (AI) use

The authors accept full responsibility for the content of the manuscript, including the disclosure of any use of AI.

No AI tools were used in the preparation of this manuscript.

### Author ORCIDs

J. Lee https://orcid.org/0000-0001-9004-8275

I.-H. Kim https://orcid.org/0000-0002-7332-0043

### Data availability

All of the data that support the findings of this study are available in the main text.
